# A qualitative study exploring the use of interpreters in a healthcare setting for children and young people seeking asylum and refugees

**DOI:** 10.1186/s12913-025-13533-8

**Published:** 2025-11-27

**Authors:** Juliana Leonardo, Bryony Hopkinshaw, Stephanie Webb, Sarah Eisen

**Affiliations:** 1 London School of Hygiene and Tropical Medicine, London, UK; 2 Children and Young People’s Division, University College London Hospitals NHS Foundation Trust, London, UK; 3 Present Address: Oxford Medical School, Medical Sciences Divisional Office, University of Oxford, Oxford, UK; 4 Hospital for Tropical Diseases, University College London Hospitals NHS Foundation Trust, London, UK; 5 Pembroke College, St Aldate’s, Oxford, OX1 1DW UK; 6 Institute of Child Health, University College London, Great Ormond Street, London, UK

**Keywords:** Children, Young people, Refugee, Migrant, Medical interpreters, Communication, Remote, In-person, Thematic analysis

## Abstract

**Background:**

Children and Young People Seeking Asylum and Refugees (CYPSAR) are increasing in number in the UK and globally. CYPSAR have many vulnerabilities which affect access to healthcare, including an almost universal requirement for language interpretation in healthcare settings. Current UK guidelines recommend in-person interpreters, but, in practice, remote interpreters are often used for pragmatic reasons.

**Aims:**

This study aimed to explore the perspectives of healthcare providers in a specialist refugee health service regarding the use of remote interpreters when caring for CYPSAR.

**Methods:**

Nine semi-structured individual interviews were performed with healthcare workers (July-August 2024). Participants were opportunistically recruited through professional networks. Interviews were transcribed and analysed thematically.

**Results:**

The key themes identified include both the positive and negative roles of interpreters in the development of patient-professional relationships, and recommendations for mitigators in this context. The significance of the interpreters role as both cultural and linguistic was discussed, as was the influence of patient age and trauma (patients’ own, and vicarious trauma for interpreters). Despite a lack of formal training, participants felt competent in using remote interpreters and that remote interpretation is generally acceptable when providing care to CYPSAR, albeit with some caveats and dependent on context.

**Conclusions:**

This work provides evidence for how best to address language barriers in healthcare provision to CYPSAR. We make recommendations to optimise effective interpretation in consultations with CYPSAR, including that provision is made for both remote and in-person interpretation.

**Supplementary Information:**

The online version contains supplementary material available at 10.1186/s12913-025-13533-8.

## Background

### The population - children and young people seeking asylum and refugees

Children and Young People Seeking Asylum and Refugees (CYPSAR) in the UK are increasing. There were 19,858 asylum applications from CYPSAR in the year ending September 2024, of whom 4,017 were unaccompanied by a parent or carer (CYPSAR-U) [1].

Whilst CYPSAR are a diverse group, this cohort are recognised to experience significant, complex and intersecting physical and mental health needs [2] which may arise from experiences prior to leaving their country of origin, during the migration journey, or on arrival to the host country. CYPSAR-U are likely to have specific additional vulnerabilities, including a lack of family support and instability around asylum status and accommodation. CYPSAR are also known to experience significant barriers to accessing appropriate care. Whilst these barriers are varied, and may be financial, cultural, institutional, digital or systems-based, the experience of a language barrier affects the majority and is identified by CYPSAR themselves as one of their biggest challenges on arrival to the UK [3].

### Importance of interpretation in health settings

Interpretation, in the context of this work, refers to the transfer of one spoken language to another, and where the word translate or translation is quoted it refers to spoken interpretation. It is well established that access to interpretation in healthcare is important for optimal care delivery [4–6]. Trained interpreters support the effective communication of patients’ concerns, symptoms and needs, and their understanding of advice and explanations of professionals.

Language barriers and non-professional interpretation have been linked to poorer quality healthcare and adverse health outcomes [4, 5, 7–10] When compared to informal, untrained interpreters, such as family or health workers, professional interpreters have been shown to reduce unnecessary treatments and diagnostic tests and increase patient satisfaction [4, 11, 12].

In a systematic review, 76% of 33 included studies (across primary and secondary care) found that language concordance improved quality of care in at least one measured outcome [13]. Moreover, a recent retrospective cohort study in Ontario, Canada, found that people who received language-concordant care experienced fewer emergency admissions and reduced mortality when compared to those who did not [14]. Use of interpreters in a paediatric surgery setting improved the patient’s medical understanding and satisfaction [15]. These findings highlight the need for accessible and adequate medical interpretation services.

Many people seeking asylum face barriers in accessing medical interpretation when presenting to health services. A retrospective qualitative study from Norway and Austria found that barriers included individual (skills and training) and organisational (funding) factors and that the use of video interpretation may circumvent these barrier [16]. There is limited and conflicting evidence on whether the mode of interpretation in health settings impacts outcomes. In a systematic review of studies with Spanish-speaking patients in the US, video interpretation provided better understanding of diagnoses when compared with phone interpretation [17]. A review by Wollscheid et al. identified three studies addressing families with children or parents with minority language backgrounds in healthcare settings. This review concluded that there was no evidence from these studies that any particular interpretation method (in person interpreter, telephone interpreter, and ad-hoc interpreter) was significantly superior in terms of outcomes for families [18]. Finally, a study into access to interpreters in a hospital setting during Covid-19 found that staff faced barriers when attempting to use in-person interpreter services, and that video services could be useful in circumventing these barrier [19].

### Interpretation with children and young people

Even when carried out in a patients’ first language, consultations with children and young people have additional complexities due to the need to adapt communication and language to the developmental stage of the child or young person. Further, the “three-way consultation” [20], with both the child and their parent/carer (where applicable), needs to be considered [21]. Interpretation with children and young people may therefore pose additional challenges [9].

### Interpretation with CYPSAR

In addition to the developmental considerations that apply to communication with all children, additional needs and vulnerabilities of CYPSAR may make communication via interpreters more complex. It is recognised that trauma can impact on communication [22], and that CYPSAR-U engagement in health services can be impacted by distrust of professionals [23, 24].

Many existing studies on interpretation in health settings, even in a paediatric context, may not be generalisable to CYPSAR. For example, a recent study of facilitators and barriers for interpreter use in European healthcare settings excluded patients seeking asylum [5], possibly resulting in under-estimation of effects of language barriers on health outcomes in this population. In addition, there is a lack of evidence that directly reflects the voices of young people themselves: parental opinions are often relied upon rather than those of children, and few studies involve CYPSAR-U [17, 18].

In a survey of European Paediatric Emergency Department staff, 60% reported that language issues were a critical barrier to care provision to CYPSAR [4]. Moreover, in the UK, language barriers have been identified by CYPSAR as a key barrier to accessing health services [3].

### Current UK policy and practice

A recent synthesis of EU and EEA policy recommendations proposed ensuring access to a professional interpreter (either in-person or remote) if limited English proficiency is anticipated by service-providers [4]. A study across the WHO European Region demonstrated the long term cost-effectiveness of the use of professional interpreters, despite the initial added cost [25]. In the UK, the Royal College of Paediatrics and Child Health (RCPCH) recommendation is to use a registered interpreter for any assessment for children and young people with a first language other than English and in-person interpretation is advised where possible [22].

A recent survey of 86 healthcare professionals, involved in performing Initial Health Assessments across 84 local authorities for CYPSAR-U, found that 90% of professionals preferred in-person interpreters. However, 51% of respondents would use an alternative option (such as a telephone interpreter) when in-person interpretation was not available, rather than rescheduling [26].

### Study aims and rationale

There is a strong theoretical basis for the impact of interpretation in influencing the quality of healthcare received by CYPSAR. Current guidelines recommend in-person interpreters, yet there is little specific evidence behind this recommendation, and it is known, anecdotally, that remote modes of interpretation are often used with this group. We aimed to explore the benefits, challenges and acceptability of remote interpretation (compared with in-person) when working with CYPSAR in a healthcare setting in the UK. Remote interpretation, as described in this study, refers to the use of professional interpreters via telephone and/or video calls. Our work, an unfunded exploratory study, focuses on experiences of healthcare providers. Healthcare providers are of particular relevance as the likely individual who determines mode of interpretation used and have multiple interactions using interpreters with patients with a range of backgrounds and needs. Thematic analysis was identified as the best method to address the objectives given the richness of data from qualitative interviewing, and the exploratory nature of the question.

## Methods

This qualitative study obtained data from semi-structured interviews with health service providers to identify key themes regarding provider experiences of interpretation services when working with CYPSAR, with a particular focus on the use of remote interpreters. The work was carried out as a student project for fulfilment of a Master’s degree. Ethics approval was obtained from the London School of Hygiene and Tropical Medicine (LSHTM) Research Ethics Committee (Project ID 30830).

### Theoretical framework

The key theoretical principle underlying this study is the importance of trauma-informed practice when working with refugee and migrant patients. Many CYPSAR have experienced traumatic events and may be further traumatised by the asylum-seeking process. The importance of patient-centred and culturally appropriate ways to build trust and engagement is widely recognised when working with CYPSAR [4, 27], due to the differences in health literacy and cultural contexts between the country of origin and where they are seeking medical attention. Quality interpretation is key to trauma-informed practice because it facilitates culturally-appropriate, clear communication, relationship building, trust and collaboration. Provision of appropriate interpreters has been identified as a key principle in trauma-informed guidelines co-developed with Albanian CYPSAR-U [28].

Given the overlapping vulnerabilities of CYPSAR, and the trauma-informed principle that care should be individualised to patients, it is important to explore the influence of interpreters in relation to this specific group.

### Setting

The study was carried out within Respond Integrated Refugee Health Service at University College London Hospital NHS Foundation Trust, UK, which is a public, National Health Service (NHS) hospital in London, UK. Respond is a free-to-use service and offers health assessments for people seeking asylum and refugees, including CYPSAR [29].

### Recruitment

Inclusion criteria were healthcare workers who either worked in Respond clinics or referred patients to Respond and regularly worked with CYPSAR using remote interpretation services.

**Fig. 1:**
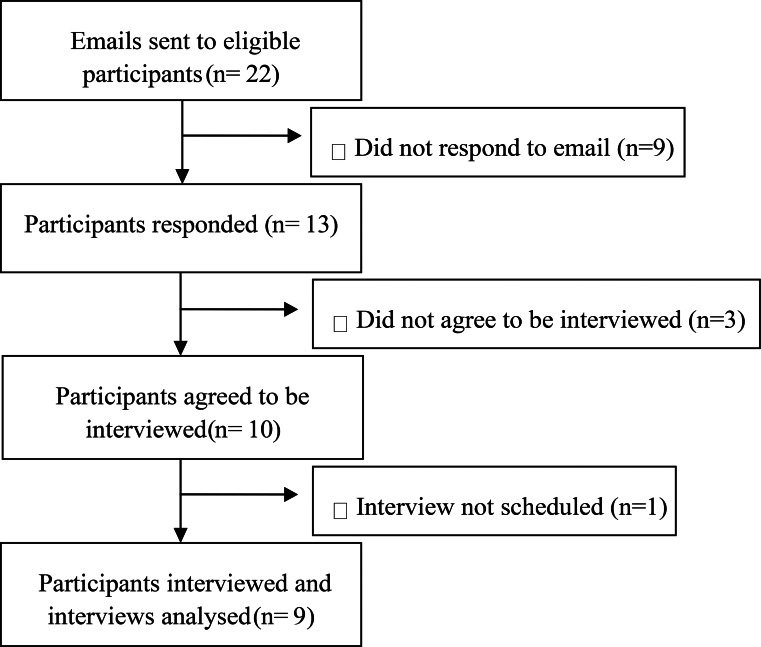
Flow diagram of participant recruitment

Recruitment emails were sent to staff meeting inclusion criteria, with nine participants recruited opportunistically based on interest and availability (Fig. 1). The interviewer had no prior relationship with any participants and all were aware that the interviewer was a student completing this project in fulfilment of a degree.

As this was an exploratory study with limited resources, we did not aim to reach or identify thematic saturation during data collection. Sample size was limited by researcher capacity and participant availability; however, it is of comparable size to other qualitative research on similar topics [6]. Participant demographics are shown in Table 1. However, participant ethnicity was omitted to ensure anonymity as Respond is a small service. All participants had experience of both remote and in-person interpretation, and none had formal training in working with interpreters.

**Table 1:** Participant demographics

Identifier	Mode of interview	Profession	Sex	Main location of practice
P1	In-person	Nurse	Female	Outpatient Service; Pediatrics
P2	In-person	Doctor	Female	Acute Pediatrics
P3	In-person	Doctor	Female	Acute Pediatrics, Infectious Disease
P4	Remote	Doctor	Female	Acute Pediatrics
P5	Remote	Nurse	Female	Outpatient Service, TB
P6	Remote	Nurse	Female	Outpatient Service, Infectious Disease
P7	In-person	Doctor	Male	Outpatient Service, Respiratory
P8	Remote	Doctor	Female	Outpatient Service, Sexual Health
P9	Remote	Nurse	Female	Outpatient Service, Infectious Disease

### Data collection

Participants were provided with a topic guide (Table 2) and participant information sheet. The topic guide included a questionnaire template for the interviews, developed by the principal investigator (JL) for the purposes of this study (see Appendix 1 in supplementary files). No pilot interviews or repeat interviews took place.

Nine semi-structured interviews were conducted with nine health service providers, by the principal investigator (JL). Interview duration ranged from 22 to 55 minutes (min) (mean = 42 min; median = 42.5 min). Interviews were conducted individually either in-person in private meeting rooms on or near the hospital site or remotely on Microsoft Teams (version 2503), depending on participant preference.

**Table 2:** Topic guide themes box

The topic guide covered health service provider’s experiences of:
• Facilitators and barriers to care using interpreters
• Their own and interpreters’ training
• Their experiences with providing care to various age groups and using different methods of interpretation
• Patient understanding and comfort
• Cultural interpretation

There was time allotted at the end of interviews to address acceptability of remote interpretation directly and discuss any relevant topics raised by the healthcare provider. The topic guide was outlined in this way to attempt to assess remote interpretation acceptability on a variety of criteria, and in different healthcare settings with different ages of CYPSAR. Interviews covered experience with CYPSAR who are accompanied by parents/carers, and those who are unaccompanied (CYPSAR-U).

Interviews were transcribed verbatim using Microsoft Office (Teams and Word, version 2503) and verified for accuracy by the principal investigator. Additionally, field notes were taken by the interviewer both during and after interviews. Transcripts were not reviewed or commented on by participants after recording.

### Thematic analysis

A thematic analysis was conducted following the methodology described by Green in The Principles of Social Research [30]. Familiarization with data was undertaken by the principal investigator after verifying the accuracy of the transcript by reading each transcript a second time. NVIVO 14 software was used to support documentation of codes. The initial deductive codes based on the pre-existing evidence base and topic guide were: Acceptability, Building Relationships, Cultural Interpretation, In-person [vs. remote] Comparisons, Further Studies [i.e. areas for future research], Barriers, Technical Barriers, Service Provider Likes, and Working with Children. Coding continued and inductive codes were created following the direction of the interviews. Each of the above groups were further coded into various sub-groupings and three new main codes (also with sub-groupings) were added: Non-verbal Communication, Service Provider Tips, and Service Provider Training. In total, 91 codes were created, which were categorised under seven eventual themes. Initial coding was carried out individually by the principal investigator, with involvement of two additional co-authors (BH and SE) in discussion and revision of eventual themes. One of these co-authors (BH) involved in discussion and revision of themes was also a participant in the research; we viewed this dual role as a form of “member reflection” 1 [31]. In addition, all participants were provided with a copy of the draft results synthesis and given the opportunity to provide further feedback. Brief positive feedback was received from four out of nine participants, with no dissent or suggested alterations to the analysis

### Reflexivity

Reflection on researcher roles and perspectives took place through reflective notes during the data collection phase, and later through discussion between co-authors.

All four researchers are white, with citizenship of high-income countries. Three identify as women, and one as non-binary/trans. The principal investigator and interviewer was a student in a health discipline at the time of the research, and the remaining researchers are doctors or medical students, all with specific professional and/or personal experience of working with people seeking asylum and refugees. As such, we consider our experiences and positionality as similar to the participants in the research study. However, our lived experience is likely to be somewhat different to medical interpreters, and very different to that of CYPSAR.

We feel that this positionality has shaped the research in a number of ways. Our pre-existing professional experience with CYPSAR shaped both the specific research questions asked, and the underlying theoretical emphasis on trauma-informed practice. Participants in the study were aware that the interviewer was a student; this awareness may have led to a tendency for participants to assume a “teacher” role during interviews, and to emphasise learning points and practical tips. In interpreting the data, the research team can be seen as “insiders” from the perspective of research participants, which is likely to be a strength both for interpretation, and for our ability to derive conclusions that are of transferable interest to other professionals working with this group of patients. However, both the research team and participants are “outsiders” from the perspective of the subjects being discussed (medical interpreters and CYPSAR). This positionality limits our critical lens when considering if, when and how CYPSAR and interpreter perspectives diverge from those of professionals. We have aimed to make this gap explicit in our interpretations.

## Results

**Fig. 2:**
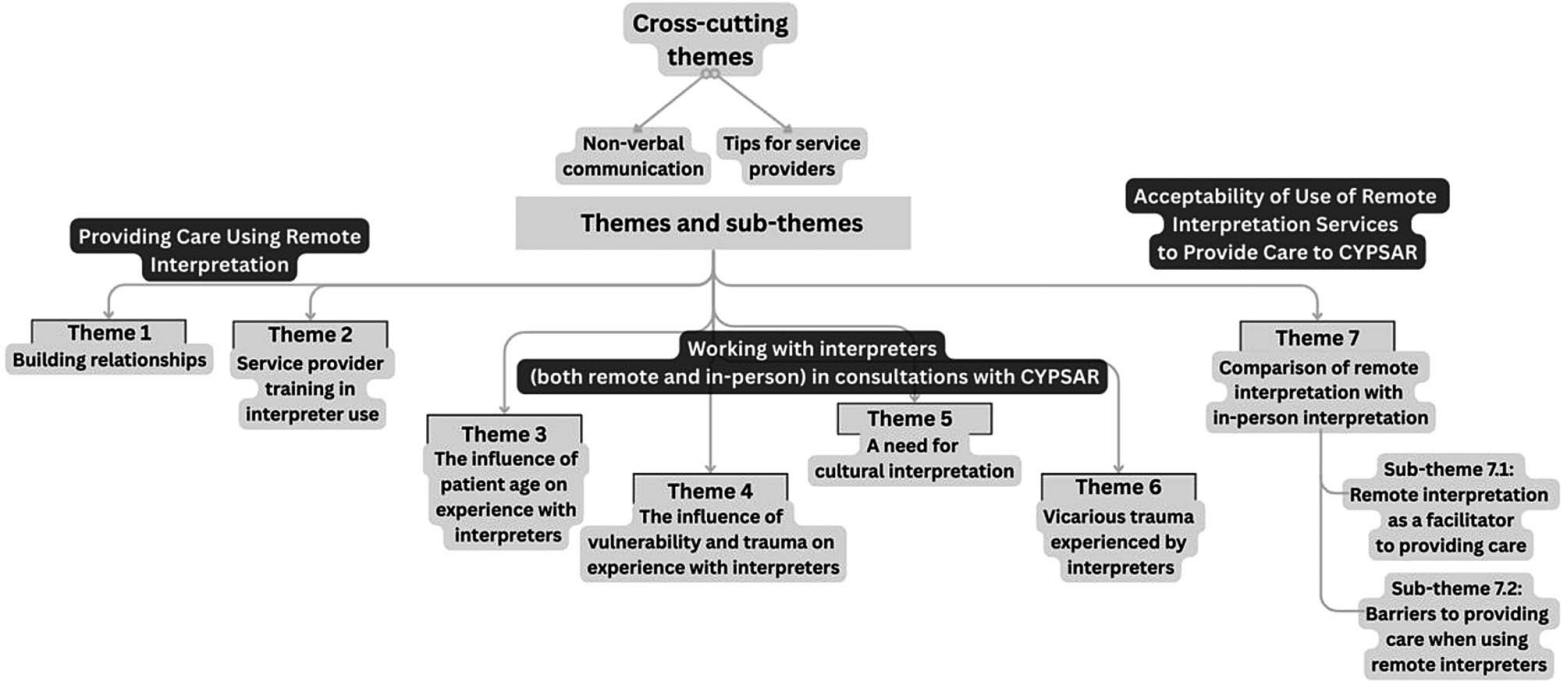
A schematic illustration of the themes and sub-themes identified during analysis

The nine participants provided insight into several topics, which were grouped under seven key themes (Fig. 2). Themes 1-2 relate to providing care using remote interpretation: building relationships; service provider training in interpreter use. Themes 3-6 relate to working with interpreters (both remote and in-person) in consultations with CYPSAR: the influence of patient age on experience with interpreters; the influence of vulnerability and trauma on experience with interpreters; a need for cultural interpretation; vicarious trauma experienced by interpreters. Theme 7 is: comparison of remote interpretation with in-person interpretation (including barriers and facilitators to care when using remote interpreters), where quotes use the phrase “face-to-face” this term refers to in-person interpretation. Throughout interviews, participants also shared, directly and indirectly, best practice recommendations for using remote interpreters; these recommendations are discussed under the relevant themes.

### Providing care using remote interpretation

#### Theme 1: Building relationships

All participants discussed building relationships between themselves and CYPSAR. This discussion included identifying the interpreter as a potential barrier in the patient-professional relationship, and techniques that were used to build patient relationships when using interpreters, for example, the professional acting as a “bridge” between child and interpreter.

Participants report using non-verbal communication to assess understanding and build relationships with service-users whilst using remote interpreters: “*99% of my perception of the empathy and trust building is coming from… smiles and eye contact and gestures that you’re having that have got nothing to do with the words*” [P2]; “*The only way I can gauge whether that young person is [understanding] is the faces that they’re pulling*,* and I do think you get quite a lot of information. From them going --Your eyebrows raised. ‘What on Earth is happening here’ versus like… nodding along*” [P3].

#### Theme 2: Service provider training in interpreter use

Although no participants had formal training on use of remote interpretation specifically, most reported learning from experience - “*you just have to learn on the job how to do it*” [P8]. Participants were largely confident in their ability to use interpretation services, but some suggested that training could be useful, and reflected that there is a lack of feedback from participants and interpreters. One participant stated: “*I feel I have a lot of experience and I hope that I’ve learned from my experience… but no one’s assessed me*,* no one’s given me feedback… the patients– haven’t told me how they find it. I’ve not heard from the interpreters how they find it working with me*,* so I don’t know. So yeah*,* I think it would be good to have training from people who actually have looked into this in more detail*” [P1].

### Working with interpreters (both remote and in-person) in consultations with CYPSAR

#### Theme 3 - The influence of patient age on experience with interpreters

An important factor in working with children and young people is age-appropriate adaptation of treatment and relationship building techniques. Participants noted that interpreters do not always seem to be trained for this: “*They speak to [the child] like an adult sometimes and don’t use the correct terminology*” [P5]. Specific difficulties with remote modes of interpretation for younger children were also mentioned: “*we use an iPad [to connect with the interpreter]*,* sometimes they’d get entirely distracted by the iPad and would be trying to press buttons and stuff… Whereas if there’s an actual person*,* they could be interacting more*” [P1].

With children, the consultation may involve multiple people (parent(s)/carers, child and clinician), and many participants discussed these dynamics. One example given was differing language proficiencies between children and their patients: “*you often have the situation where the kid speaks English because they learn it so quickly… the interpreters for the parent … The kid understands the question before the parent does. So you have to really kind of think carefully about what you’re asking… I would want them to be involved in answering the questions*,* but… I don’t want them to have to be the adult in the situation*” [P2].

#### Theme 4 - The influence of vulnerability and trauma on experience with interpreters

Participants noted witnessing distrust and discomfort in this population, perceived to be likely related, in part, to their vulnerability within the legal system of the UK and/or the country and community of origin. This observation was noted to make it harder to build trust with health-service providers, and is a potential specific issue with remote interpretation: “*I think one of the big issues*,* particularly with unaccompanied asylum seekers is… the understanding… that the clinic is not connected to the Home Office… I think that– that fear exists hugely whatever the setup*,* but probably when you have a remote interpreter it makes it… that bit harder*” [P2].

Participants also noted that trauma can impact on a consultation in multiple ways, and that it can be hard to unpick whether a lack of understanding is a result of mis-interpretation, a patient’s trauma, or both: “*It’s really hard to know how much they understand*,* and I think a lot of that is*,* as I said*,* like health literacy*,* but also thinking about… previous trauma and what people have been through*,* you know*,* and their retention*,* their memory*,* their concentration*,* all those things that are gonna be affected by kind of their*,* their histories and stuff*” [P9].

Participants commented on how the lack of time with patients, and the fact that they may often only be seen once, inhibits ability to build relationships and gain trust. “*Yeah*,* I think in my experience*,* [the CYPSAR] tend to be a little bit more guarded compared to the adults… it’s difficult to know how to build that rapport sometimes and you don’t have a lot of time*” [P8].

#### Theme 5 - A need for cultural interpretation

Sometimes, as a result of the specific needs of this population, interpreters will act as cultural interpreters. Most participants could recall at least one instance in which a remote interpreter strayed from interpreting verbatim between service provider and service user to provide a cultural insight or clarification: “*she [the interpreter] was kind of like contextualizing… in their culture*,* which was good*” [P4]. An extension of this cultural mediation is interpreters stepping in to inform the health worker that a certain topic or word was off limits in the patients’ culture: “*…sometimes they [the interpreter] will make it explicit*,* … I definitely had interpreters say like… that interaction I had with the teenager who didn’t want to give us a stool sample and I was trying to pry… they could have really translated*,* but it was a cultural problem…”* [P2]. Several participants gave examples of this.

However, participants also gave examples of where interpreters sharing culture with patients could be problematic, leading to both patients and interpreters being worried about stigma. “*How does an interpreter feel talking to another [person from the same country] about sex? I hope they’ve been trained*,* but because of the shame surrounding it*,* that might be a difficult conversation. So then what are they saying that changes what you’re saying*,* and what’s the cultural differences and the cultural nuances there? I have no idea and I’d be interested*” [P1]. One participant gave an example in which a young person muted the interpreter to ask a question pertinent to sexual health via Google Translate.

Despite interpreters sometimes acting to bridge cultural gaps, a lack of healthcare workers’ shared cultural context with patients was reported as an important barrier when using interpreters. This observation was a particular issue with sensitive or potentially stigmatised topics, such as sexual and mental health. For example, participants discussed how the concept of “mental health” may mean different things when interpreted: “*Lots of these countries… mental health is either a taboo or they’re not talking about it…. So then how are they receiving the translation? Do they have any context? So if the translator directly says mental health*,* what does that translation mean to that person? Does the translator say*,* are you crazy… I don’t know how that is interpreted…*” [P1]. One participant gave examples of steps that they would take to try and mitigate some of these cultural gaps: “i*f I’m talking to Kurds or Afghans about sexual health…I’ll caveat it and I’ll say*,* ‘I know that you’re Muslim and you’re not thinking about this*,* but’. So that they feel*,* like*,* acknowledged. But also*,* I know that… they’re going to school and they probably are thinking about it….So you need to equip them*,* but you don’t want to shame them…*”[P1].

#### Theme 6 - Vicarious trauma experienced by interpreters

Participants recognized that interpreting traumatic accounts of past abuse, the immigration process, and personal histories is likely to take a toll on remote interpreters. These worries led participants to also question what kind of support and trauma-based care training interpreters received, as well as worrying that some interpreters may have similar traumas in their background. “…*there’s the sort of like vicarious trauma that is not only for us*,* but also for the interpreter that he’s interpreting for us… We don’t have any way of checking on them and… we’re not sure whether they have any support and they may be of similar background as well*,* but we we don’t know*” [P4].

### Acceptability of use of remote interpretation services to provide care to CYPSAR

#### Theme 7: Comparison of remote interpretation with in-person interpretation

Participants identified both advantages and disadvantages of in-person and remote interpretation. For remote interpretation, ease of access was highlighted: “*it’s easier to get hold of [the interpreter] and at short notice*” [P8]. For in-person interpreters, the benefit, particularly for younger children, of non-verbal communication was mentioned.

All participants stated their belief that remote interpretation is acceptable in their clinics and specialties; however, some caveats were mentioned. While some preferred remote interpretation, some felt that whilst acceptable, it is not ideal. Others felt that it is acceptable only in certain circumstances, acceptable pending more research into interpreter training and patient preferences, or acceptable simply because of practical/logistical constraints. “*It all comes down to accessibility*,* doesn’t it?… remote… makes complete sense for A&E. I just wonder whether face-to-face feels more useful in a clinic where– where you have longer and you’re building a rapport and you can predict which languages you’re actually going to need to have interpreters for*” [P3].

There were differing perspectives on what modality may be preferred by patients, including the influence of trauma on preference. For example, one participant said: “*I suppose the patients like some to be face-to-face. Sometimes it’s easier*,* you know*,* because they – you can see the eye contact. You can see the body language*” [P5]. Several participants gave scenarios in which remote interpretation could be preferable, for example: “*It’s almost less intimidating having to talk about quite intimate things when it’s just a voice*,* a nameless voice on the phone*” [P8] or “*Often the time it takes to get people online is very*,* very fast. I think sometimes it’s difficult to have a fixed start time to a clinic. Some people turn up late*,* sometimes you overrun*” [P7].

#### Sub-theme 7.1: Remote interpretation as a facilitator to providing care

Participants identified a range of ways in which remote interpreters facilitate their provision of healthcare, such as technological benefits including ease, effectiveness, and the improvements of interpretation technology over time: “*the idea that you can almost immediately have somebody on the line who can speak the same language it’s kind of crucial because… in A&E … you need to quickly kind of adapt”* [P3]. Remote interpretation was also felt to facilitate care, including the importance of interpreters in providing comfort to patients, the high quality/training of interpreters themselves, as described by one participant: *“There’s this one interpreter that comes to mind… he shows so much respect to the patient… I understand some of like the words that he’s saying to the patient and he’s so lovely”* [P1].

#### Subtheme 7.2: Barriers to providing care when using remote interpreters

A barrier for health service providers in providing care with remote interpreter use is a perceived lack of training of the interpreter, or providers feeling under-trained themselves. One participant said that they felt repetition and re-phrasing can be part of a trauma-informed approach, however interpreters can sometimes be frustrated by this method. One healthcare provider felt it was part of their role to *“help [the interpreter] understand*,* you know that we might need to repeat the question a few times. We might need to phrase it differently. That’s OK”* [P9]. Perceived and/or confirmed misinterpretations were also frequently mentioned as barriers to providing care, including the issue that both sides may not be aware if a misinterpretation is occurring: “*we just have to trust [in the interpreter]”* [P1].

The loss of visual information acts as another barrier for health service providers and interpreters: “*And yeah*,* I think it’s just easier face to face*,* especially those they’re very sensitive conversations to be having as well… the interpreter can see the body language of the patient*,* so they they can see if they’re getting a bit upset*,* give them a breath. Then we can come back and reconvene again. But. a telephone interpreter can’t see that*” [P5]. It should be noted here that most participants commonly used telephone interpretation opposed to video interpretation resulting in this loss of visual information.

There were significant logistical and technological barriers which participants experienced, including the unavailability of languages, long waits for the interpreter, being connected to an interpreter of the wrong dialect, clerical delays, restrictions caused by booking policies, and interpreter shift changes during consultations. Difficulty hearing due to technology was also a common barrier mentioned: *“Technical difficulties as well not hearing very well*,* not like– voice cracking*,* line dropping*,* and cutting off*,* things like that that when you are in the middle of a consultation about the trauma*,* it may be impacting the conversation”* [P4].

### Summary

These seven key themes are inter-related, and we note commonalities between many of the themes, in particular the role of non-verbal communication, and the way that providers gave recommendations for working with interpreters with CYPSAR. These topics are discussed further as “cross-cutting” themes below.

## Discussion

This study identified seven key themes perceived by healthcare staff as important when working with interpreters with CYPSAR. These include ways in which relationships are built during consultations with interpreters, a need for training in working with interpreters, patient-related factors that influence experience with interpreters (age, vulnerability and trauma, and culture), concerns regarding vicarious trauma of interpreters, and comparing remote interpretation with in-person. Non-verbal communication, and provider tips for working with interpreters, recurred in relation to all key themes, and are therefore discussed here as cross-cutting themes.

The majority of these themes align with existing literature about barriers and facilitators when working with interpreters, and important considerations when working with CYPSAR. However, we note that the final theme - comparing remote with in-person interpretation - suggested a nuanced picture in which in-person interpreters may be preferred in certain circumstances, and remote in others. This advice differs from current UK guidelines (e.g [22]. which suggest a blanket preference for in-person interpreters where possible, however, it is more in-line with current Australian guidance [32]. These finding indicate that international guidelines should be regularly crossed-checked to achieve optimal care for CYPSAR globally.

### Theme 1. Building relationships

Participants discussed relationship-building in the context of working with CYPSAR as both vital to building trust and impeded by remote interpretation. The importance of rapport and trust building is supported by a recent literature review about providing care to people seeking asylum in high-income countries [33], and is a core principle of trauma-informed practice [34]. That (remote) interpreters were seen by some participants as a barrier to trust and rapport suggests that - to mitigate this effect - additional attention to relationship building may be important when interpreters are used. Participants themselves frequently made recommendations to support building of trust and rapport. Some participants used non-verbal communication to this end by using open and friendly body language and gestures and using body language cues from CYPSAR to gauge understanding.

### Theme 2: service provider training

Participants had not received formal training in interpreter use and suggested that care provision using remote interpretation could be improved by increased access to training. There is existing evidence that using trained rather than ad hoc interpreters improves the quality of care [4, 11, 12]. Although a need for increased training of healthcare professionals in the use of interpreters is commonly cited in studies of existing training levels (e.g [11]), a 2024 systematic review into training programmes for working with medical interpreters showed that there is limited evidence of improved clinical outcomes [35]. Nevertheless, these programmes were reported to be well received by clinicians. Many studies which report on training programmes on interpreter use focus on healthcare students or early career professionals, however, our study suggests that the appetite for provider training in working with interpreters remains (or is perhaps stronger) even among clinicians with significant pre-existing experience in interpreter use. Participants referred to both increased training for themselves and clarification regarding training policies for interpreters as key factors in improving confidence in remote interpretation services. Further research into how interpreters perceive the training level and need of the clinicians they are interpreting for would elicit interesting future results.

### Theme 3 - The influence of patient age on experience with interpreters

While most participants trained to specialize in paediatric medicine or nursing, none were formally prepared for incorporating an interpreter into a dynamic in which there are often parents or carers present during consultations. A recent report on using interpreters with children recommended that family dynamics with interpreters should be incorporated into future training [9]. Building trust is important and can be aided by allowing family or carers to stay in the room [33], however, participants described instances in which it may be important to talk to children and young people alone. These differences in strategy are obviously context-dependent and need to be anticipated and navigated carefully by health service providers on an individual basis.

### Theme 4 - The influence of vulnerability and trauma on experience with interpreters

Past trauma can impact CYPSAR in a number of ways, as identified by participants. The effects of trauma on the brain can make understanding and processing information more difficult [22]. Moreover, past traumas, especially regarding medical care may make trust and relationship building harder. This issue is especially concerning in this population, as individuals seeking asylum who do not trust the professional who is consulting with them have been shown not to return for medical advice or treatment for themselves or their children [33].

The impact of vulnerability and trauma is likely to interrelate with a number of other themes. For example, it was mentioned by participants that the impact of trauma on the consultation is particularly significant when working with CYPSAR-U. CYPSAR-U are usually adolescents, and the recognition of this developmental stage (linking to theme 3) is likely to be related to the way they are impacted by trauma, as well as to challenges around building rapport and relationships (theme 1).

### Theme 5 - A need for cultural interpretation

Differences in cultural background can result in misunderstandings and difficulty accessing health services [36, 37]. Participants all independently recognized instances in which they had perceived a need for cultural interpretation, or in which care was impacted by a culturally-rooted misunderstanding between the health service provider and CYPSAR. In line with our findings, previous studies have identified linguistic and cultural factors as a barrier to healthcare for people seeking asylum, highlighting the deficit of cultural competence in some healthcare professionals as a predominant issue [6, 36]. The importance of cultural sensitivity was also recognised as a key factor in trauma-informed practice in work co-developed with Albanian CYPSAR-U [28].

Cultural competency is a theme where the need for further insight from CYPSAR themselves and from interpreters is particularly great. For example, participants described incidents where interpreters added cultural context to their interpretation. Whilst this intervention was broadly felt to be helpful by the healthcare provider participants, it would be of interest to know how interpreters feel about having to take on this role, and how it is experienced by patients. There were suggestions in the data that there are certain contexts - for example sexual health - where patients having shared culture with interpreters may be perceived negatively by the patient. This example suggests a need for nuance and individualisation around recommendations for remote vs. in-person interpreters. This area in-particular warrants further research, owing to its relevance in increasingly diverse urban areas and health systems.

### Theme 6 - Vicarious trauma experienced by interpreters

The trauma experienced by the young person may also personally impact the health-service provider and interpreter [6, 38]. The concerns raised by participants in this study about the impact on interpreters aligns with some existing research on vicarious trauma in interpreters. Numerous studies in varied settings have reported on this issue, such as a study in 1999, surveying Red Cross Interpreters in Geneva, where 66% had frequent painful memories from their sessions [39], and a 2015 survey of Australian interpreters where four in five experienced psychological distress [40], and finally, by a study in the US where medical interpreters reported vicarious trauma [41]. A recent systematic review investigating the mental health of interpreters working in mental health care services for refugees found that interpreters had heightened stress and anxiety and secondary stress reactions to their consultations [42]. The healthcare providers in this study recognised the impact that various trauma may have on interpreters who work for the service, and the need to document these experiences and provide adequate debriefs and care for interpreters [43]. Our findings suggest that not only is there a need for support for interpreters, but healthcare providers also have a desire to know about the support available to interpreters, and how considering the interpreters own needs can be incorporated appropriately into consultations.

### Theme 7 - Comparison of remote interpretation with in-person interpretation

It remains debated whether in-person or remote interpretation (telephone/video) services are preferable in healthcare settings, both in terms of quality of care and patient and provider preference. Many participants also described their experiences with remote interpreters through comparison to other formats of interpretation such as in-person and informal. We found that, in a specialist service for CYPSAR, remote interpretation is acceptable to health care providers, and that, in specific (often sensitive) circumstances, is felt to have advantages over in-person modes.

A recent literature review, focussed on European healthcare settings, investigated professional interpreter use by identifying facilitators and barriers to availability, accessibility, acceptability, and quality of care [5]. The UK-based studies included identified numerous ‘availability’ barriers including “lack of immediate availability, interpreters [being] unavailable outside of working hours, number of interpreters unable to meet demand, and language not covered/unavailable” [5]. Remote interpretation may overcome some of these availability barriers and decrease financial burden, however this benefit could be at the expense of other aspects of quality, such as loss of visual interactions. Interestingly, a recent study of refugee mothers with limited English proficiency in Australia found that while in-person interpreters were universally preferred, professional remote interpreters were also recognized as important assets for communication with and within the hospital [44]. This finding highlights the need to investigate both provider and consumer experiences of interpretation.

In our study, technological aspects of remote modes were described both as facilitators to care (for example ease of access, particularly in settings where speed is important such as emergency departments) and as barriers (for example signal cutting out). Whilst lack of specific training for remote interpreters in working with CYPSAR was cited as a barrier to quality care, examples were also given of particularly positive experiences with certain interpreters. Although a lack of body language cues was described as a barrier to care when using remote interpreters and a reason to use in-person modes for more sensitive consultations, it was also described that some young people may prefer the anonymity of a telephone interpreter for intimate topics.

It is important to note that most participants in this study used telephone (rather than video) when accessing remote interpreters which is relevant in relation to certain described downsides - such as lack of body language - as well as potential positives such as a feeling of anonymity.

Current UK practice guidelines recommend that in-person interpretation is preferred where possible [22]; however, findings from this study, albeit only including healthcare providers, suggest a more nuanced picture in which in-person interpreters may be preferred in certain circumstances - such as complex discussions - and remote in others, such as ad hoc/unplanned consultations, and in line with patient preference for certain sensitive topics such as sexual health. Our findings are in agreement with those from Australia, where a more nuanced approach to remote versus in-person is recommended [32].

### Cross-cutting theme - non-verbal communication

This study found that non-verbal communication was a key cross-cutting theme impacting consultations between CYPSAR individuals and healthcare providers within our service. Non-verbal communication was more frequently identified as a facilitator to care by health-service providers, than as a barrier to interpretation. Specifically, within theme 1 (building relationships), we found that non-verbal cues were integral to building rapport. Within theme 7 (remote interpretation as a facilitator to providing care), the importance of in-person interpretation and non-verbal communication, especially for young people, in a clinic setting where time is less limited, was reported. A systematic review on effective healthcare communication with children and young people found that non-verbal cues may indicate specific meanings to young people [45]; this finding is important in the context of our work with CYPSAR individuals where cultural context may also impact the interpretation of meaning. Moreover, a narrative synthesis exploring communication between people seeking asylum and healthcare professionals in primary care found that non-verbal cues were key in creating a comforting space to build rapport [46]. These findings are in agreement with our study that non-verbal communication is essential for rapport and highlight the need for more work to promote non-verbal communication as a driver in quality care for CYPSAR.

The experience of the Covid-19 pandemic provided a new method of communicating with patients through telemedicine, and research on non-verbal communication between healthcare providers and patients emerged. A scoping review of telemedicine consultations between healthcare professionals and patients, found that the most prevalent barriers were difficulty in recognising facial expressions, body positioning, and gestures [47]. These are areas where cues may be missed by CYPSAR using remote interpreters and emphasises the need for medical interpreter services tailored to the context of the clinical setting. Our study demonstrated that healthcare professionals sometimes felt that remote interpreting may be less appropriate for CYPSAR in outpatient clinics than in the emergency department. This finding underlines the necessity of future work into how non-verbal communication impacts the quality of relationships and services provided specifically to CYPSAR, particularly from the perspective of CYPSAR themselves.

Non-verbal communication cross-cuts into theme 2 (healthcare provider training) and theme 3 (influence of patient age on the experience with interpreters). Healthcare professionals who work in paediatrics are trained in communication with people of different ages, but the interpreters used do not necessarily have this training and expertise. In the context of remote interpretation, the visual cues relating to the patient’s age may be lost, and hence, the interpreter may use communication that is inappropriate. There is limited previous research into the role of non-verbal communication between interpreters and CYPSAR, and the influence of age and service provider training. Our study provides further insight into the need for appropriate training and specialised interpreter services for this demographic.

Non-verbal communication is also integral to how people communicate traumatic events when using English as a non-native language [48]. A qualitative study into cross-cultural communication in asylum seekers, found that different themes, emotions, and contexts were communicated when using their native language in comparison to English. This work feeds into our findings in theme 4 (influence of vulnerability and trauma on experience with interpreters) and theme 5 (need for cultural understanding), where non-verbal cues surrounding trauma may be lost during interpretation, and thus the ability to understand and treat the trauma is bypassed. In relation to context-specific preferences for remote vs. in-person interpreters, consultations where traumatic experiences are to be communicated effectively and/or treated is likely to be an example where in-person interpreters are preferable and important.

### Cross-cutting theme - tips for providers

Practical recommendations for providers were shared by participants in relation to many themes, particularly around relationship building with CYPSAR, and on adapting to patient specific factors such as age, vulnerability and culture. These recommendations are summarised in Table 3.

**Table 3:** Practice pointers box

Practice pointers box
• Provide choice to patients: e.g. phone vs. video, gender of interpreter (where possible).
• Clarify confidentiality, including that information will not be shared with immigration services
• Set the scene with the interpreter: explain who is in the room; set expectations (e.g. for full, not abridged interpretation); consider giving the interpreter a warning regarding potentially distressing topics.
• Communicate in ways that can be interpreted clearly: speak in short segments, signpost the conversation, avoid euphemisms, ask a single question at a time.
• Recognise that certain concepts (e.g. “mental health”) may not be interpreted directly and consider breaking it down into more specific concepts (e.g. stress, poor sleep).
• Be conscious and adaptive to the multiple factors that can influence a patient’s experience with an interpreter including cultural backgrounds, age, gender, dialect and prior experiences.

### Strengths and limitations

A strength of this study is the depth and breadth of experience with CYPSAR among participants. Nurses and doctors working in various parts of hospitals and clinics, including those working primarily with children and those seeing children only as part of their practice, were selected to understand a range of perspectives. As researchers we reflected throughout our on our positionality and the potential bias this imparts on the study. Still, while we practised reflexivity as part of our research methodology, it is likely that both the researchers’ and participants’ own cultural, linguistic and migration backgrounds, will have influenced their perspectives on the topics discussed. However, due to the small sample size, the demographic data collected and presented was limited to protect participant anonymity. We note that the majority of our participants were female or assigned female at birth and acknowledge that the limited diversity of the investigators and participants reduces the generalisability to people and teams comprised of varied cultural and ethnic backgrounds.

This research comprises a single-site study within a specialist service for CYPSAR. Whilst the extensive experience of participants in working with CYPSAR meant they had in-depth insight into the research questions, their perspectives may not be transferable to practitioners who only occasionally work with CYPSAR, particularly in relation to training and experience in working with interpreters. Allied health professionals were not interviewed, nor were students (some participants were professionals in training) this could result in further decreased transferability especially with regards to Theme 2. Further, our service explores a broad range of health conditions but is not a specialist mental or sexual health service; experience of interpretation may differ in these specific sensitive contexts. We note, however, that many of the key themes -for example, a need for cultural interpretation, and vicarious trauma of interpreters- are in line with existing evidence derived from other services, including mental health services.

Regarding transferability, it is important to note that in this study, the majority of participants were referring to telephone (rather than video) interpreters, when discussing “remote” interpretation, although participants did not always differentiate. In the existing literature on interpreter mode, the evidence relating to video interpretation is often described separately to that relating to telephone interpretation (e.g [17], and the fact that our data mixes discussion of these modes is a limitation in terms of contextualising the findings within this literature. However, this perceived limitation may also reflect the real-world experience of healthcare providers in which different remote modes are not strongly differentiated.

In this study, we did not aim to reach or assess for thematic saturation. This limit was due to the small nature of the study as a student project, as well as to the relatively limited pool of eligible staff. Instead, we aimed for the project to be exploratory; identifying key themes of interest that inform future research. This limit may have led to themes such as improving health equity and safety of care through the use of interpreters not being identified. This results differs slightly from other studies in this area that highlighted how using interpreters correctly and safely may reduce harms in similar population [7, 8, 49]. We note that if thematic saturation has been reached, we may have elicited similar themes in our analysis, and that future work exploring these themes is required.

The most significant limitation of this study is the absence of the voices of interpreters, and, most importantly, of CYPSAR. This avenue did not form part of this small study due to time and funding limitations but is a key component of planned forthcoming work, based, in part, around key themes identified here.

## Conclusions and recommendations

We have identified important and key themes regarding interpretation in healthcare encounters with CYPSAR. The impact of interpretation on relationship building, role of training, and patient factors which may influence the interpreting experience are significant. Our findings suggest that, while remote interpretation presents some additional challenges when compared with in-person interpretation, it is acceptable to service providers when working with CYPSAR, and in some contexts may even be preferable. This study identifies a number of practical recommendations for working with interpreters in consultation with CYPSAR, derived from experienced healthcare providers.

The experiences and observations shared by participants can serve to guide further studies into the use of remote interpretation in clinical settings for CYPSAR. There is a significant need to explore the perspectives of service-users [25]. Interpreter perspectives would be particularly pertinent in relation to our themes of service-provider training, vicarious trauma of interpreters, and the cultural context in interpretation. It would be helpful to explore contexts which can guide individualised recommendations for mode of interpretation, including patient-specific factors (such as age, cultural background, or accompanied/unaccompanied status) as well as the subject matter of the consultation.

Interpreters will continue to play a key part in providing quality, trauma-informed services for CYPSAR. We recommend that services not only ensure access to trained interpreters for all children and young people who need them but also allow for flexibility, so that both access to and training for remote and in-person modes are available when needed or preferred.

## Supplementary Information

Below is the link to the electronic supplementary material.

Supplementary Material 1

## Data Availability

Data is freely available on request from the corresponding author and if accepted we will happily share raw interview files as additional files with consent forms or on a public repository.

## Footnotes

1 BH joined on as a co-author months after the completion of the first draft of this work. She was not involved in the project in any way outside of her participation as an interviewee until this time. JL and BH first met in the context of an interview for this project. She was not involved in thematic analysis nor did she have access to non-anonymized interviewee data.

## Abbreviations

CYPSAR: Children and Young People Seeking Asylum and Refugees
CYPSAR-U: Undocumented Children and Young People Seeking Asylum and Refugees
LSHTM: London School of Hygiene and Tropical Medicine
NHS: National Health Service
RCPCH: Royal College of Paediatrics and Child Health
