# Supplementary material for: A qualitative study exploring the use of interpreters in a healthcare setting for children and young people seeking asylum and refugees
Source: BMC Health Serv Res. 2025 Nov 27;25:1547. doi: 10.1186/s12913-025-13533-8 (PMC12661805; doi:10.1186/s12913-025-13533-8)
Supplement: Supplementary file 1 — Supplementary Material 1 [file 12913_2025_13533_MOESM1_ESM.docx]

# Appendix 1: Topic Guide

## Semi-Structured Interviews with Health Care Providers Topic Guide

Introduction: My name is [NAME] and I am conducting these interviews in fulfillment of an MSc in Public Health. My project is entitled “A Qualitative Investigation into the Use of Interpreters in RESPOND Clinics for Minor Asylum-Seeking People” and has an aim of investigating the facilitators and barriers for interpreters interacting with minor asylum seekers in the RESPOND integrated refugee health service. Project objectives are listed below. These interviews should take roughly 45 minutes to complete.

Objectives: To understand health-service provider perspectives on providing the best possible service to minors while using interpreters. To determine the acceptability of using remote interpreters to both health-service providers and health service-users at clinics.

### Opening

I. What do you believe is important to understand about working with remote interpreters with this population?

Topic Areas

1. Technical Facilitators
   1. What do you like about remote interpretation services?
   2. How do remote interpretation services aid you in your job?
2. Technical Barriers
   1. What are some barriers (if any) to remote interpretation services?
   2. Are there any problems that you face while working with remote interpretation services?
      1. Please describe why yes or no
3. Training
   1. Do you feel you have adequate training to work with remote interpretation services?
      1. Please describe why yes or no
   2. What is your perception of remote interpreter training to be interpreting with a vulnerable population and in a clinic-setting?
4. Age Groups
   1. Have you ever worked with different age groups?
      1. If yes, which age groups?
   2. What is your perception of the differences between different age groups (if any) you have worked with?
5. Comparisons to In-Person Interpretation Services
   1. Have you ever utilized an in person interpretation service?
      1. If yes, are there any perceivable differences between the in-person and remote services?
      2. If no, why not?
6. Unique Characteristics of Working with Minors
   1. What is unique about being a health service provider to minors?
      1. Under 5 years of age, under 10 years of age, under 15 years of age, under 18 years of age?
   2. Do remote interpretation services seem to have unique effects on younger service-users?
      1. Please describe why yes or no
   3. How do young patients typically respond to sensitive questions such as sexual history and mental health concerns?
7. Level of Understanding on Health-Provider Side
   1. What is your perception of patient needs, questions, and descriptions being interpreted back to you well?
8. Perceived Level of Understanding for Patient
   1. What is your perception of patients being able to understand what you said after remote interpretation services?
   2. What is your perception of interpretation of things like empathy and trust?
9. Perceived Comfort of Patient
   1. Do patients appear comfortable with remote interpreters?
10. Cultural Interpretation
    1. Do remote interpreters offer any “cultural interpretation” in your experience? (this could include improving your understanding of cultural norms, attitudes, behaviors)

Closing

1. Is the use of remote interpreters acceptable for minor asylum seekers?

A. Are there any topics we have not covered that influence this opinion?

1. Are there changes that you believe could make remote interpreter use better/acceptable?
2. What do you believe should be studied further in this topic?
3. Is there anything we haven’t covered that you would like to discuss?

Thank you for your time, participation, and insight.
